# Automated Segmental Analysis of Fully Quantitative Myocardial Blood Flow Maps by First-Pass Perfusion Cardiovascular Magnetic Resonance

**DOI:** 10.1109/access.2021.3070320

**Published:** 2021-04-01

**Authors:** MATTHEW JACOBS, MITCHEL BENOVOY, LIN-CHING CHANG, DAVID CORCORAN, COLIN BERRY, ANDREW E. ARAI, LI-YUEH HSU

**Affiliations:** 1 National Heart, Lung, and Blood Institute, National Institutes of Health, Bethesda, MD 20892, USA; 2 Department of Electrical Engineering and Computer Science, The Catholic University of America, Washington, DC 20064, USA; 3 Circle Cardiovascular Imaging Inc., Calgary, AB T2P 3T6, Canada; 4 British Heart Foundation Glasgow Cardiovascular Research Centre, University of Glasgow, Glasgow G12 8QQ, U.K.; 5 West of Scotland Heart and Lung Centre, Golden Jubilee National Hospital, Glasgow G81 4DY, U.K.; 6 Radiology and Imaging Sciences, Clinical Center, National Institutes of Health, Bethesda, MD 20892, USA

**Keywords:** Cardiovascular magnetic resonance, myocardial perfusion imaging, myocardial blood flow, image segmentation

## Abstract

First pass gadolinium-enhanced cardiovascular magnetic resonance (CMR) perfusion imaging allows fully quantitative pixel-wise myocardial blood flow (MBF) assessment, with proven diagnostic value for coronary artery disease. Segmental analysis requires manual segmentation of the myocardium. This work presents a fully automatic method of segmenting the left ventricular myocardium from MBF pixel maps, validated on a retrospective dataset of 247 clinical CMR perfusion studies, each including rest and stress images of three slice locations, performed on a 1.5T scanner. Pixel-wise MBF maps were segmented using an automated pipeline including region growing, edge detection, principal component analysis, and active contours to segment the myocardium, detect key landmarks, and divide the myocardium into sectors appropriate for analysis. Automated segmentation results were compared against a manually defined reference standard using three quantitative metrics: Dice coefficient, Cohen Kappa and myocardial border distance. Sector-wise average MBF and myocardial perfusion reserve (MPR) were compared using Pearson’s correlation coefficient and Bland-Altman Plots. The proposed method segmented stress and rest MBF maps of 243 studies automatically. Automated and manual myocardial segmentation had an average (± standard deviation) Dice coefficient of 0.86 ± 0.06, Cohen Kappa of 0.86 ± 0.06, and Euclidian distances of 1.47 ± 0.73 mm and 1.02 ± 0.51 mm for the epicardial and endocardial border, respectively. Automated and manual sector-wise MBF and MPR values correlated with Pearson’s coefficient of 0.97 and 0.92, respectively, while Bland-Altman analysis showed bias of 0.01 and 0.07 ml/g/min. The validated method has been integrated with our fully automated MBF pixel mapping pipeline to aid quantitative assessment of myocardial perfusion CMR.

## I. INTRODUCTION

First pass gadolinium-enhanced cardiovascular magnetic resonance (CMR) perfusion imaging allows for fully quantitative assessment of myocardial blood flow (MBF) and has proven to have a diagnostic value for coronary artery disease as well as myocardial ischemia [1]–[6]. Research has been successful in fully automating the process of MBF quantification at the pixel level [5], [7]. These and other works demonstrated that fully automatic MBF pixel maps provide similar blood flow values to positron emission tomography [8], are capable of diagnosing coronary artery disease [5], [9], [10], and have high repeatability [11]. However, these analyses were performed based on manually segmented MBF maps according to the American Heart Association (AHA) segment model [12]. In other words, users were required to manually draw regions of interest (ROIs) around the myocardium and identify the right ventricle (RV) insertion point to divide myocardial sectors for segmental analysis. Not only is this process tedious and time-consuming, but it is prone to errors.

The research and development process of automatic segmental analysis of fully quantitative MBF pixel maps is relatively new, and only a handful of works have recently been published on automated myocardial segmentation. Scannell *et al*. [13] employed deep learning-based methods for myocardial segmentation based on 175 CMR perfusion studies. The segmentation process was split into four steps, each trained separately. Their data was split into three groups: 135 training data, 10 validation data and 30 testing data. The first step identified the frame of peak left ventricle (LV) enhancement. Second, a bounding box around the heart region was defined using this time frame. Third, the myocardium was segmented from the bounding box. Fourth and finally, the RV insertion point was detected and used to split the myocardium into different sectors.

Xue *et al*. [14] presented a deep learning approach for automatic MBF quantification and segmental analysis of myocardial perfusion imagery. Their method was trained on a dataset of 1825 perfusion scans from 1034 patients. An additional 200 perfusion scans from 105 patients were used as an independent testing dataset. Before segmentation, the perfusion series were pre-processed to transform them into gadolinium concentration series. Then the transformed training data were split further into training and validation sets consisting of 87.5% and 12.5% of studies, respectively. A U-net semantic segmentation architecture based Convolutional Neural Network (CNN) was trained to segment the myocardium, RV and LV. The RV region was used to detect the RV insertion point based on AHA segment model. Unlike Scannell, which operates on images after scanning, Xue’s work is integrated with a dedicated imaging sequence [7] for image acquisition and reconstruction to allow in-line pipelined processing of the MBF maps as soon as the images are acquired.

In this work, we present a fully automatic method for perfusion MBF pixel map segmental analysis. The automated pipeline begins by locating the RV insertion via landmark detection, followed by segmentation of the myocardium from the dynamic perfusion images via region growing, edge detection, principal component analysis, and active contours. to segment the myocardium, detect key landmarks, and divide the myocardium into sectors appropriate for analysis. This result is used as a starting point for the myocardial segmentation of the MBF perfusion maps, using similar methods, before the region is finally divided into myocardial sectors with the detected RV insertion point.

## II. METHODS

### A. IMAGE ACQUISITION

A retrospective dataset of 247 clinical CMR perfusion studies was included in the present study. All studies were performed under procedures and protocols approved by the institutional review board of the National Heart, Lung and Blood Institute, and all subjects gave written informed consent (ClinicalTrials.gov Identifier: NCT00027170) over a four-year period. Demographic information of the patients is shown in Table 1. In these studies, gadolinium-enhanced CMR perfusion imaging was performed on two 1.5T scanners (Siemens Healthcare, Erlangen, Germany) with a saturation recovery steady-state free precession dual-sequence technique [15]. Three short-axis slice locations of the heart at base, mid, and apex were acquired at every RR interval over 60 heart beats during breathhold.

Gadolinium-DTPA (Magnevist, Berlex Laboratories, Wayne, NJ, USA) was administered (0.05 mmol/kg) at 5 ml/s during vasodilator stress and rest perfusion imaging followed by a saline flush. Typical imaging parameters for the myocardial perfusion image series included: 90° composite saturation preparation pulse, 50° flip angle, 90 ms inversion time, 1.2 ms echo time, 2.3 ms repetition time, 8 mm slice thickness, 360×270 mm field of view, 128 × 80 acquisition matrix, 256 × 192 image matrix after interpolation, and parallel imaging factor of 2 [16]. During each perfusion acquisition, a low-resolution arterial input function (AIF) image series was also acquired using a fast low-angle shot sequence with a separate saturation pulse. Typical imaging parameters for the AIF series were: 8° flip angle, 5.0 ms inversion time, 0.7 ms echo time, 1.3 ms repetitive time, 10 mm slice thickness, and 64 × 48 acquisition and image matrix size. The dedicated AIF series was specifically designed to maintain linearity of signal intensity within the LV, providing accurate AIF measurements. At the beginning of each perfusion imaging, two proton density weighted images were also acquired without saturation preparation pulse which are used for surface coil intensity correction.

### B. IMAGE PROCESSING

**FIGURE 1.**
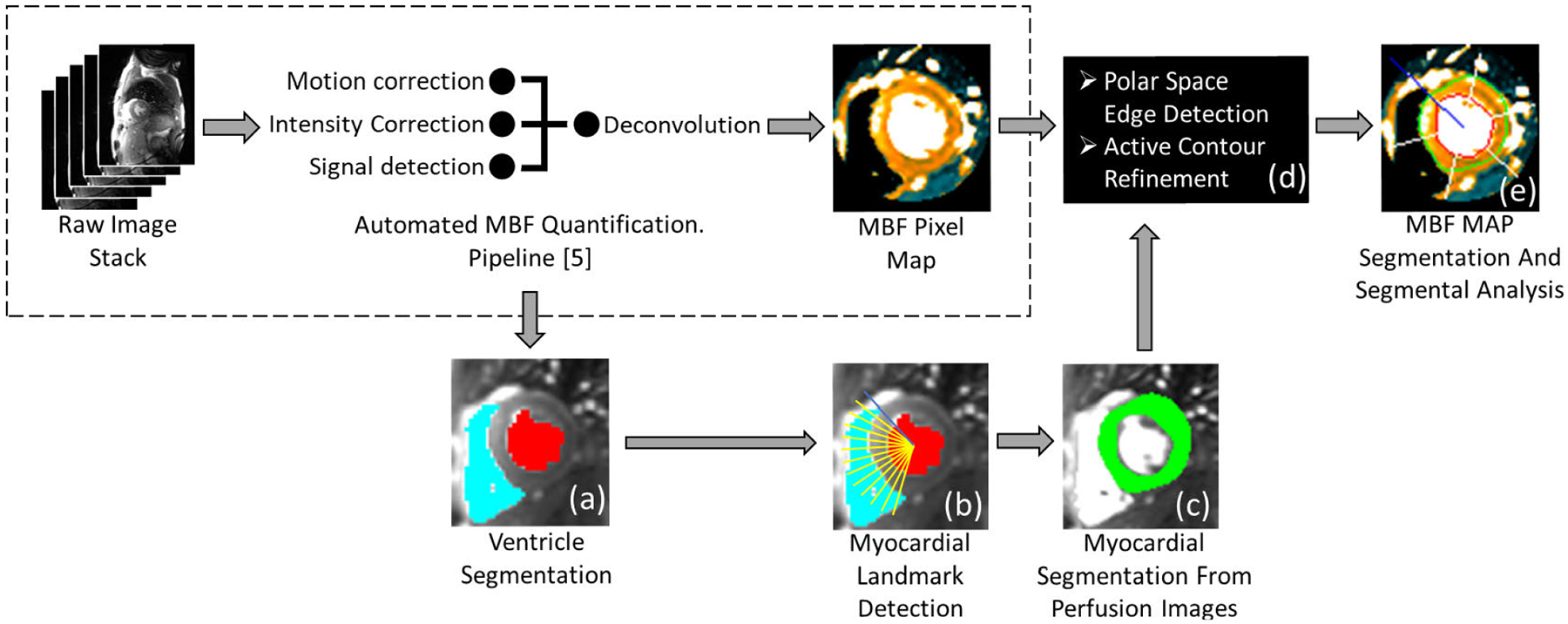
Image Processing Pipeline. An outline of the proposed methods for automated MBF pixel map segmentation, labeled from (a) to (e). They are integrated with the fully automated pixel-wise MBF quantification processing pipeline we developed previously (dashed box). More details of steps (c) can be found in Figure 2; steps (d-e) are further detailed in Figure 3.

An outline of the proposed image processing pipeline is shown in Figure 1. This pipeline is an extension of our automated pixel-wise MBF quantification pipeline previously presented in [17] and [5]. A non-rigid body image registration technique based on optical flow computation was first applied to correct for motion artifacts that may exist in the image series [18]. Our previous work [17] described a fully automated method of measuring the AIF from CMR perfusion images. The AIF was extracted only from the basal slice for subsequent use in MBF quantification at each slice location. Our previous work [5] described a fully automated pipeline which includes a model constrained deconvolution technique to estimate pixel-wise MBF values in ml/g/min. The main additions allowing for automated segmental analysis, which this section will focus on, are the automation steps for RV insertion point landmark detection, perfusion image myocardial segmentation, MBF map myocardial segmentation, and myocardial sector definition. The remaining steps in Figure 1 have been previously described in [17] and [5].

While our goal is to perform myocardial segmentation on the MBF map, this process is more robust if the myocardium is segmented from the perfusion images first and then propagated to the MBF map for further refinement. Because the LV and RV cavities can be detected more reliably from the perfusion image series via different contrast enhancement phases, they provide a reliable anchor space for the initial myocardial segmentation.

#### 1) LANDMARK DETECTION

To follow the segmental analysis recommendations of the AHA [12], the angle of the anterior junction point of the RV and LV, hereto referred to as the RV insertion point angle, must be identified. The RV and LV were segmented from the basal slice using a multi-level standard deviation threshold, weighted voting scheme, and independent component analysis similar to our previous work [17]. The angle is detected automatically by measuring the angles between the boundary points of the RV and the center of the LV. The image is then rotated to a common reference model, with the RV points to the left of the LV. From this orientation, the RV insertion point will be the pixel on the RV wall that has the smaller counterclockwise angle from the origin at the LV midpoint, as seen in Figure 1(b). The RV insertion point angle is measured from this and will be used for segmental analysis. The same angle is used on all three image slices, but the angle’s origin will be re-centered onto the LV of each slice, as detected in the following section.

#### 2) MYOCARDIAL SEGMENTATION FROM PERFUSION IMAGES

**FIGURE 2.**
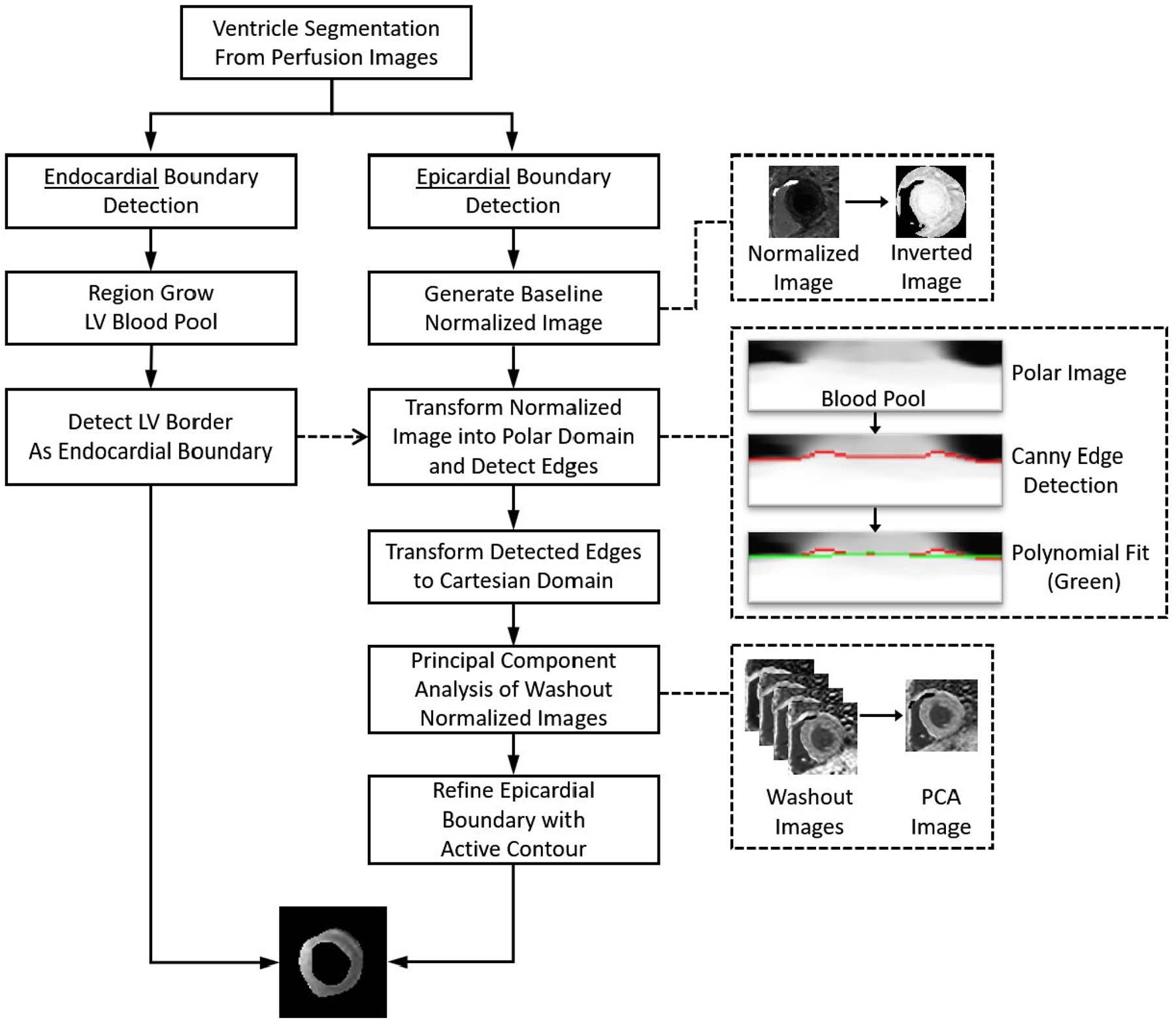
Myocardial Segmentation from Perfusion Images. An outline of the automated image processing steps for CMR perfusion image segmentation. This is part of the proposed automated pixel-wise MBF quantification processing pipeline as shown in Figure 1.

The myocardium is detected on each slice location sequentially starting from the basal and proceeding to the apical slices. Figure 1(a–c) outlines example result of the process, while the individual steps are more precisely outlined in Figure 2. In the basal slice, the LV boundary, Figure 1(a), is refined using region growing from the initial LV cavity mask using the frame of peak LV intensity. A convex hull is applied also ensure that any the papillary muscles present are included with in the LV boundary. Selecting the enhanced LV boundary as the endocardial boundary ensures the exclusion of the papillary muscles. The epicardial edge of the myocardium is detected from a time-signal intensity normalized image series. This is distinct from 2D image normalization which, in general, sets the maximum intensity value to 1, the minimum to 0, and appropriately scales the intermediate values to the same range. Instead, we normalize each pixel individually based on its intensity range over time. That is, the pixels’ maximum value over the time series is set to 1, while its minimum is set to 0, and intermediate values are scaled appropriately to this range. This pixel and time-based normalization highlights the relative contrast enhancement timing of each pixel rather than its absolute signal intensity magnitude. This is useful in cases with perfusion defects which do not enhance with contrast as much as healthy tissue. The normalization amplifies this small enhancement to the same level as healthy myocardial enhancement to provide better contrast for segmentation. Using this normalized series, a rough estimate of the edge is detected from a baseline intensity image, in which the myocardium and LV are dark while the background tissue is relatively bright. We chose the baseline image because there is generally a larger relative intensity difference between the mid-level background and the dark baseline myocardium than there is to the mid-level background and the perfusing myocardium. This image is transformed into the polar domain centered on the LV, and Canny edge detection is applied [19]. The longest continuous edge is extended around the myocardium using a polynomial fitting and selected as the initial epicardial edge and is transformed back to the cartesian domain. Finally, this edge is refined using an active contours algorithm [20] performed on a contrast enhanced image during the washout phase. This image is reconstructed using principal component analysis (PCA) [21] to remove redundant information and extract the most prevalent myocardial contrast enhancement information. We construct the principal component images using the images after the peak contrast enhancement (i.e. during the washout phase) and select the first principal component image that contains the primary information of the images. This first principal component image has the effect of noise reduction and signal intensity contrast improvement for better myocardial boundary detection.

The same process is applied to the mid and apical slices, with one extra step: the LV and RV must be re-located by cross correlating the heart region pixels with the ventricles signal measured from the previous slice. The pixel with the highest coefficient of correlation is selected as a seed point, from which the remainder of the ventricle is region grown; this step is similar to the LV refinement performed on the basal slice. The LV in all slices is region grown based on the peak intensity frame as measured from the basal slice. It is possible that the ventricles, particularly the RV, may not be visible in the apical slice. If the RV is not detected, the processing continues without it. However, the processing for myocardial segmentation is aborted if the LV cannot be located.

#### 3) MBF MAP SEGMENTATION AND SEGMENTAL ANALYSIS

**FIGURE 3.**
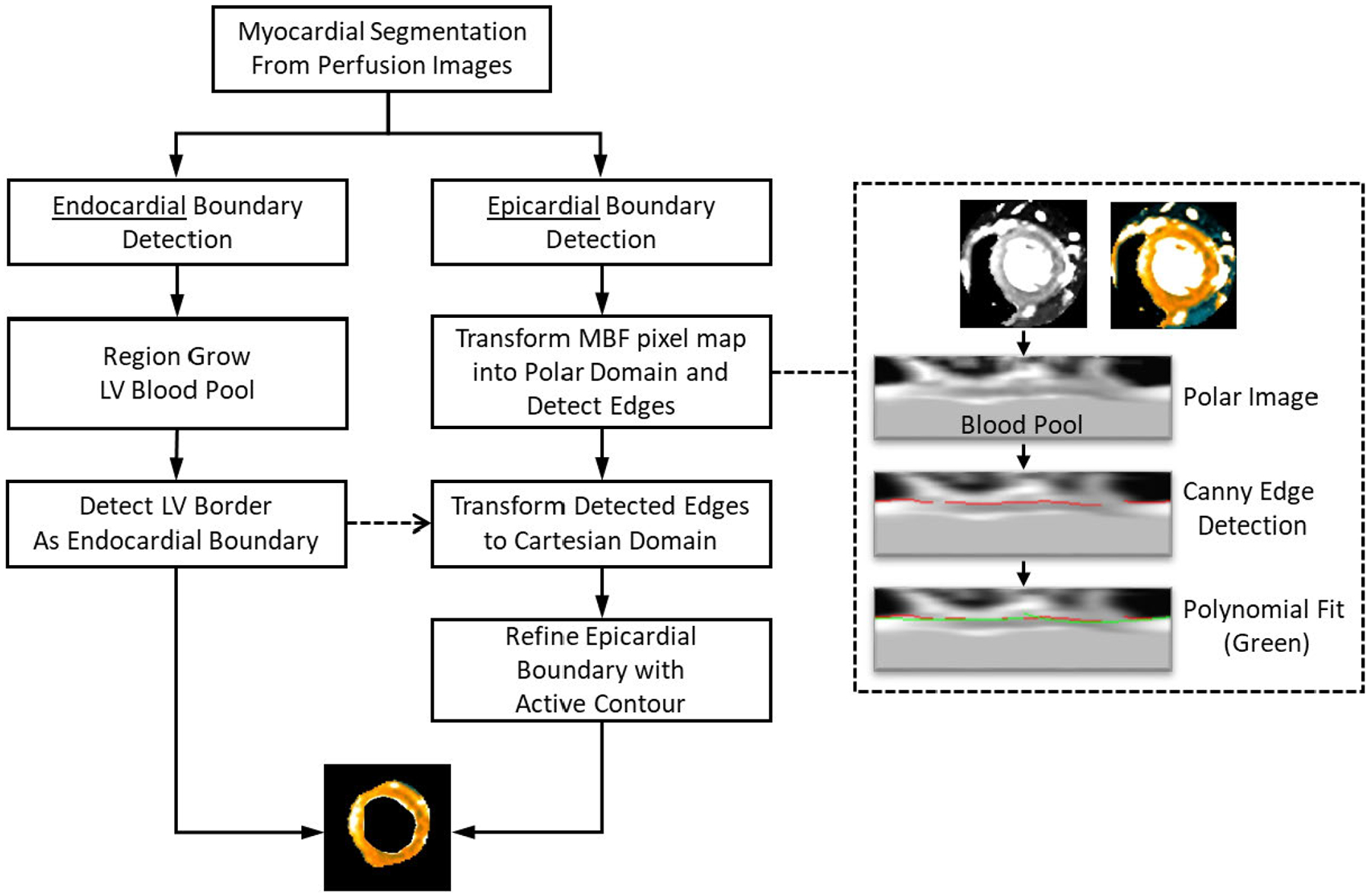
Myocardial Segmentation from MBF Maps. An outline of the automated image processing steps for MBF pixel maps segmentation. This is part of the proposed automated pixel-wise MBF quantification processing pipeline as shown in Figure 1.

After the initial myocardial segmentation on perfusion images is completed, the myocardial region is propagated to the fully quantitative MBF maps for further refinement. This is performed using similar steps as the previous myocardial segmentation, but now taking advantage of the increased contrast provided by the MBF maps and the myocardial segmentation from the perfusion images. Sample results are shown in Figure 1(d–e) while Figure 3 outlines the process in more detail. The endocardial boundary is region grown from the LV, now more distinguished by the blood pool’s extremely high MBF value. The epicardial boundary is also more easily differentiated from the non-perfusing tissue in the MBF maps using the same polar transform edge detection followed by active contours algorithm refinement.

With the myocardium delineated, the detected RV insertion point angle is used to define an arbitrary number of sectors for myocardial segmental analysis on each slice, using the center of the LV as the angle’s origin. Average sector-wise MBF can be automatically measured and reported, providing fully automatic segmental analysis of the MBF pixel map.

### C. QUANTITATIVE EVALUATION

To validate its performance, the proposed automated method was tested for segmental analysis of MBF maps from all three slices of the rest and stress series of the 247 studies. The method was implemented and executed using custom image analysis software developed in Interactive Data Language (IDL, Harris Geospatial Solutions, Melbourne, Florida). To provide a comparison, the same dataset was also manually processed by a trained CMR expert tracing the endocardial and epicardial borders of the myocardium on the MBF map using custom interactive image analysis software, also implemented in IDL. The ROIs were placed to exclude papillary muscles from the endocardial border, and anomalously bright regions along the epicardial border (likely right ventricular blood, fat, or coronary arteries). The RV insertion point was also manually selected to define six myocardial sectors, following the recommendations of the AHA [12], for segmental analysis. Sector-wise MBF and MPR measured from the automated and manual segmentation of the three slices were compared.

Agreement between the myocardial segmentations of the proposed method and the manual reference was measured using Dice coefficient and Cohen Kappa statistic. The agreement between the epicardial and endocardial borders was measured by the Euclidean distances between the automated and manual borders. The average sector-wise MBF and MPR calculated within the myocardial sectors generated by the proposed and the manual methods were compared using linear regression analysis, Pearson’s correlation coefficient, interclass correlation coefficient, Bland-Altman plots, and non-parametric Mann-Whitney U test to determine statistical significance. Results are shown as mean ± standard deviation (SD).

Additional quantitative metrics for the overall assessment of the automated vs. manual segmentation are provided in the appendix. These include commonly established statistics of accuracy, sensitivity, specificity, positive predictive value (also referred to as precision), volume overlap error, and relative volume difference [22]. Further border distance measurement metrics include, Hausdorff distance, Mahalanobis distance and the average symmetric surface distance [22].

## III. RESULTS

**FIGURE 4.**
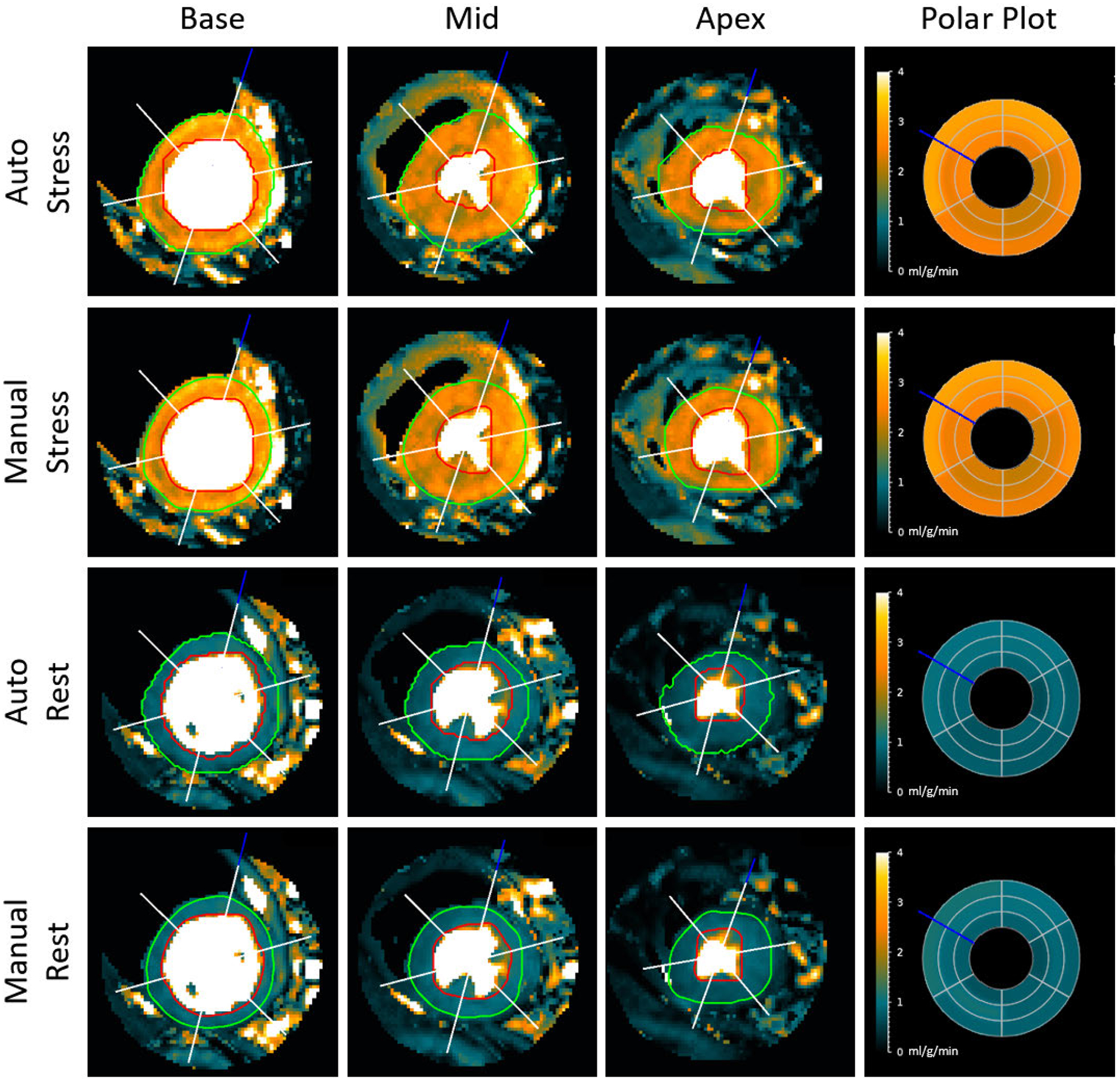
Myocardial segmental analysis of a healthy heart. The left three columns show automatic and manual segmental results on the MBF map images for a healthy volunteer. The red ROI indicates the endocardial boundary, and the green the epicardial boundary. The blue line indicates the angle of the RV insertion point, and the white lines show the delineation of six sectors for sector-wise analysis. MBF polar plots in the right column show the average flow values (in ml/g/min) from each sector. The sectors from outermost to innermost are the base, mid, and apex slices.

**FIGURE 5.**
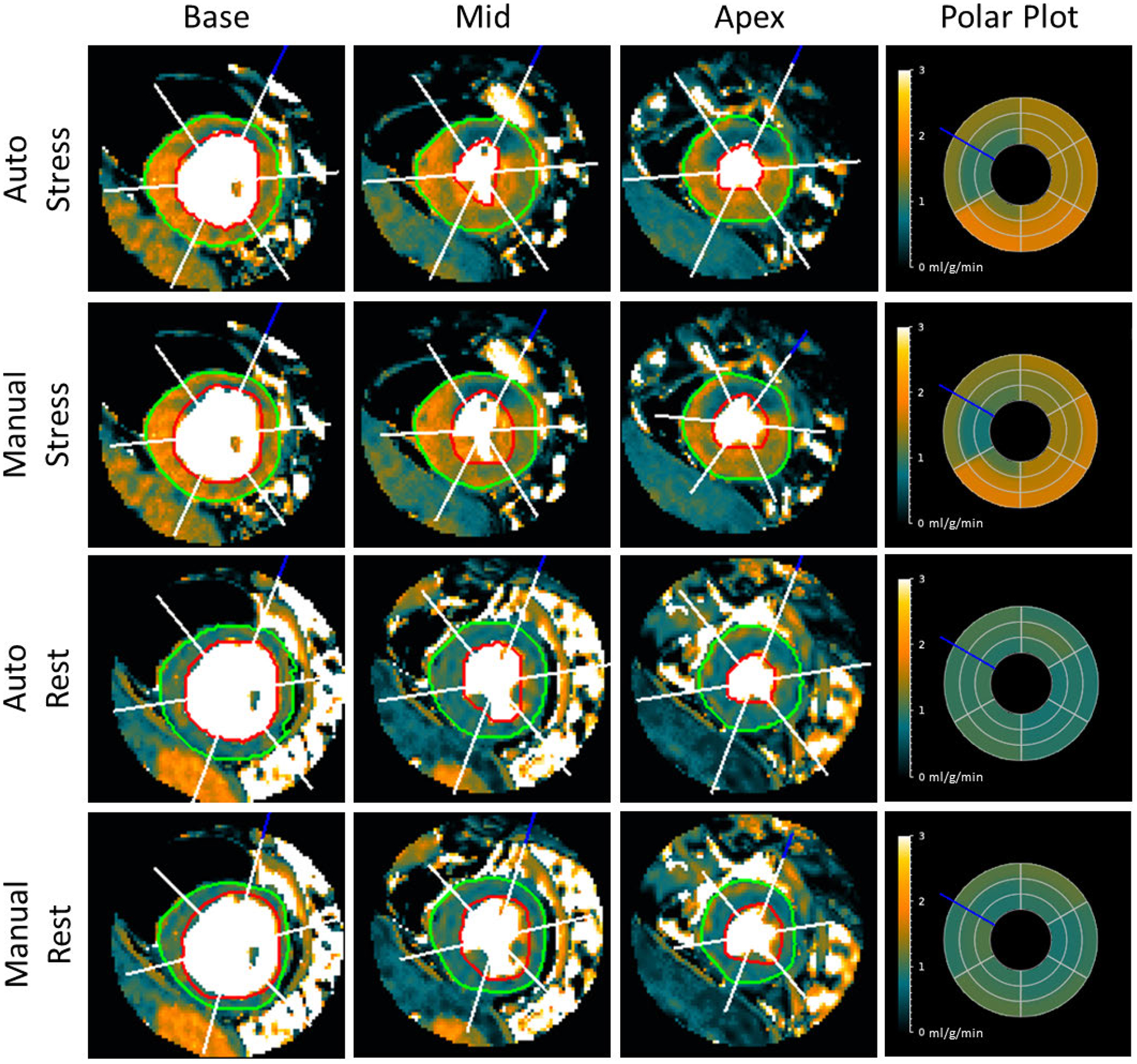
Myocardial Segmentation from a heart with a perfusion defect. Automatic and manual segmentation results on the MBF maps with a perfusion defect from a patient with suspected coronary artery disease. MBF polar plots show the average flow values (in ml/g/min) from each sector.

Of the original 247 clinical studies, 2 were excluded due to poor image quality preventing reliable MBF quantification. The proposed automated method successfully processed 243 of the remaining 245 clinical studies, yielding a 98.38% success rate and 486 scans of validation metric data. Figures 4 and 5 provide example results of MBF map segmentation from a healthy volunteer and a patient with a perfusion defect, respectively. In Figure 4, one can see that both the automatic method and the manual reference depict similar areas, though the manual segmentation favors borders that include slightly less myocardial tissue to ensure exclusion of the LV, RV, and surrounding tissue. Despite this, the sector plots show that sector-wise average blood flow is nearly identical between manual and automated measurements for both rest and stress. Figure 5 shows similar agreement on a case with a regional perfusion defect. The stress sector plots of manual and automated origin both show values consistent with regional perfusion defects, which is supported by the visual impression on all three stress slices.

Table 2 summarizes the area agreement metrics for the MBF map segmentation in each slice and the overall average, which showed good agreement between manual and automatic methods. Dice scores of at least 0.8 were achieved in 85.8% of the total 1458 MBF maps, and 99.5% of all maps had Dice scores of at least 0.6. Table 3 compares the Euclidian distances between the manual and automated segmentation of epicardial and endocardial borders, showing very good, overall sub-pixel level agreement in the tested dataset. These distances were calculated as the average of the root-mean-square distance of each boundary pixel on the automatically segmented boundary to the nearest pixel on the manually defined boundary. Table 4 and 5 show the area agreement and Euclidian distances, respectively, but separated into rest and stress series. This analysis shows that the proposed method performs similarly for both series, though perhaps slightly better on rest series. For the selection of the RV insertion point, the absolute angle difference averaged only 3.19 ± 4.16° between the manual and automatic methods.

**FIGURE 6.**
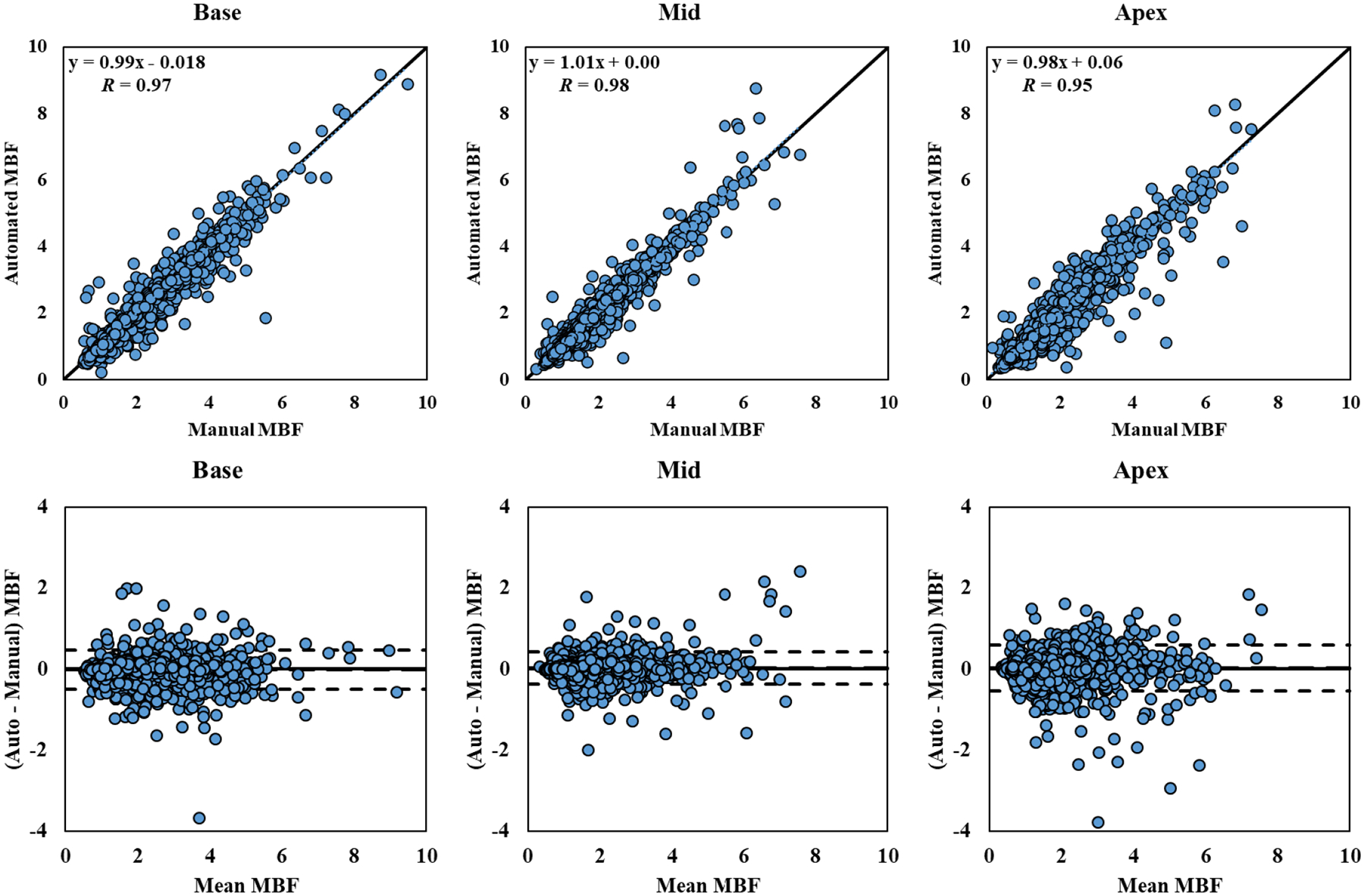
Sector-wise MBF Correlation and Bland-Altman Plot. Correlation and Bland-Altman plots of automatic and manual sector-wise MBF values (in ml/g/min) for the 243 studies processed, separated by different slice locations. The dotted blue lines in the first row represent the trend of linear regression. The dashed lines in the second row represent the bias (automated – manual) and limits of agreement (mean ± 1.96 SD).

Figure 6 displays the linear regression and Bland-Altman analyses of the automatically and manually measured sector-wise MBF values from the segmental analysis of all 243 studies analyzed, separated by slice, including the limits of agreement (mean ± 1.96 SD). Sector-wise MBF values from automatic and manual segmentation correlated well, with an average Pearson’s coefficient of 0.97 (*p* < 0.001), interclass correlation coefficient of 0.97 (95% confidence interval: 0.97; *p* < 0.001), and an *R*^2^ value of 0.94 in all three slices. Bland-Altman analysis showed minimal bias overall (0.01 ml/g/min) and SD (0.25 ml/g/min) between the two methods. Automatic and manual sectorized MBF values differed on average by 0.14 ± 0.30 ml/g/min (*p* = 0.189) for base, 0.11 ± 0.17 ml/g/min (*p* = 0.630) for mid, and 0.17 ± 0.24 ml/g/min (*p* = 0.236) for apex.

**FIGURE 7.**
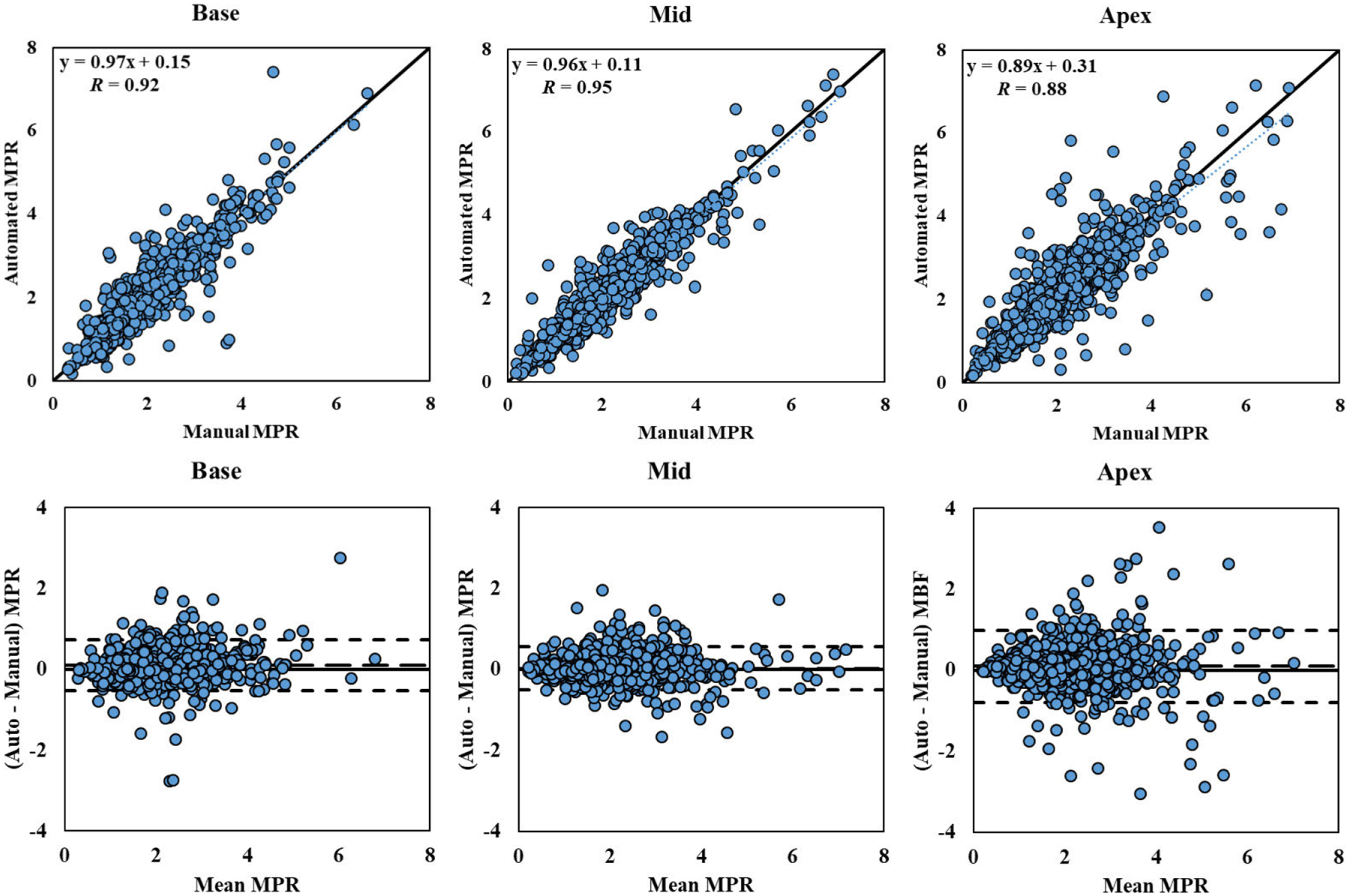
Sector-wise MPR Correlation Plot and Bland-Altman Plot. Correlation and Bland-Altman plots of automatic and manual sector-wise MPR values for the 243 studies processed, separated by different slice locations. The dotted lines represent the trend of linear regression. The dashed lines represent the bias (automated – manual) and limits of agreement (mean ± 1.96 SD).

Figure 7 displays the linear regression and Bland-Altman analyses of the automatically and manually measured sector-wise MPR values of all 243 studies, separated by slice, including the limits of agreement (mean ± 1.96 SD). Sector-wise MPR values from automatic and manual segmentation also correlated well, with a Pearson’s coefficient of 0.92 (*p* < 0.001), interclass correlation coefficient of 0.92 (95% confidence interval: 0.91 – 0.92; *p* < 0.001) and an *R*^2^ value of 0.84. Bland-Altman analysis showed a slightly larger bias (0.07) and SD (0.36) in MPR than MBF between the two methods. Automatic and manual sectorized MPR values differed on average by 0.21 ± 0.26 (*p* < 0.001) for base, 0.17 ± 0.21 (*p* = 0.433) for mid, and 0.29 ± 0.36 (*p* = 0.001) for apex.

Additional quantitative evaluation of our method based on independent datasets acquired with different imaging protocols are provided in the appendices as an external validation. Average execution times for the proposed method on myocardial image segmentation and landmark detection was approximately 6 seconds, and myocardial segmentation on the MBF map took approximately 2 seconds per slice on a desktop computer with an Intel Core i7–6950 3.0 GHz processor.

## IV. DISCUSSION

We have presented a fully automated method for segmental analysis of fully quantitative CMR perfusion MBF pixel maps. It has been evaluated on a large clinical dataset and processed 243 of 247 studies, with Dice scores of at least 0.8 in 85.8% of the maps. Automatic myocardial segmentation of MBF pixel maps were in good agreement with the manually segmented reference myocardial regions and RV insertion landmarks. Further, automatic sectorization and sector-wise MBF and MPR measurements were shown to agree with manual reference measurements. These results indicate that the proposed method agrees with manual reference standards both in terms of the segmented area, sector definition, and region derived MBF measurements. By providing the AHA recommended segmental analysis while eliminating the tedious need of manual myocardial segmentation and sector definition, the proposed method speeds the extraction of quantitative medical information for a patient, and hopefully increases the throughput and clinical utility of fully quantitative CMR perfusion imaging.

Our rest vs. stress Euclidian distance metrics show that the proposed method has a slightly better performance on the rest series. This increased performance is likely due to a greater uniformity of the rest perfusion images and MBF maps that improves the myocardial region detection especially for the endocardial boundary. Our MBF and MPR comparisons also show that the proposed method performed better on the basal and mid slices than on the apical slice. The apical slice presents larger partial volume effects in the perfusion images which can result in residual motion issues after motion correction that can challenge even manual segmentation. Further, the RV blood pool, which is helpful in defining a portion of the epicardial boundary for the LV myocardium, is not always visible in the apical slice. These issues resulted in lower area and border agreement, as well as lower correlation between MBF and MPR measurements with the manual reference for the apical slice. However, the Bland- Altman plots show no systemic bias in the automated apex slice measurements, despite a larger dispersion. In our statistical comparisons of automated vs. manual sector-wise MBF and MPR values, there are larger errors in the MPR than in the MBF comparison among all three slices. Because the MPR is a ratio measurement of two MBF values, any discrepancy in one of the two MBF value can lead to a greater discrepancy in the MPR measurement due to the division operation.

Of the original 247 study dataset, two studies were excluded from the quantitative evaluation. Both were due to image quality issues that made MBF maps results ambiguous for manual segmentation. From the remaining 245 studies, the proposed method was able to process all but two. One failure was due to an extremely small LV blood pool in the mid slice, despite that both basal and apical slices went through the automatic segmentation successfully. The other was due to a failure to correctly segment and differentiate the LV and RV. As mentioned during the method description, detection of the LV in all three slices is paramount to locating the myocardium. Examination of Figures 4 and 5 show that the automated method is more inclusive than the manual reference, often including more edge pixels that the more conservative manual segmentation excluded. This extra inclusion however did not adversely affect sector-wise MBF values, resulting in minimal and non-significant difference. These minor differences had a slightly more significant effect on the MPR measurement, likely due to the division operation previously discussed.

Previous CMR perfusion image segmentation research has mainly focused on segmenting the myocardium from the perfusion image series. Spreeuwers and Breeuwer [23] detected the ventricles by region growing from regions of maximal intensity. The LV boundary was used as the endocardial boundary, while the epicardial boundary was segmented using a contrast-ratio maximizing snake, initiated upon the RV border nearest to the LV. To highlight the myocardium, this process was performed on a difference image between pre- and post-contrast arrival maximal intensity projection. When tested on 30 image series and visually analyzed, the method successfully segmented 86.7% of them. Gupta *et al*. [24] proposed training active appearance models on 50 pre-labeled images to model a mean target object, and variations using features of region shape and texture. The completed model was tested on the basal and mid slices of 18 perfusion studies and was deemed successful on 83.3% of them. The semi-quantitative measurement of perfusion upslope was not found to be significantly different from manual segmentation. Tarroni *et al*. [25] presented a semi-automated segmentation method requiring a user-defined seed point to be selected within the LV cavity. A region-based level set technique based on the normal distribution of noise in the blood pool and myocardium was used to detect the epicardial boundary. The endocardial boundary was located with classic edge-based level set methods, and a user selected the RV insertion point for sector definition and measurement of semiquantitative metrics. The segmentation was visually evaluated as accurate. Sector-wise measurements of signal intensity showed good signal-to-noise ratio, frame by frame intensity agreement, and semi-quantitative metrics were like a manual reference. Further, the semi-quantitative metrics were shown to have similar diagnostic accuracy to a quantitative coronary angiography reference. Beache *et al*. [26] developed a method using level sets combining probabilistic shape priors, Gaussian modeling of intensity probability distribution, Potts models, and Gibbs potentials. This segmentation was performed on each image, and then each region was deformed to be consistent with a reference region over time. Validation tests showed the method was superior to two other generic shape-based segmentation methods in 24 datasets.

Some researchers have also investigated the feasibility of segmenting the myocardium from perfusion images to extract the region-wise or sector-wise time signal intensity curves for perfusion analysis. Adluru *et al*.’s method [27] detected the epicardial boundary from the LV, intensity thresholding a polar image centered on the pixel with maximal weighted intensity variance, presumably within the LV. The endocardium was then segmented by an evolving ring guided by a level set algorithm, initiated a set distance out from the detected endocardium. This was performed in five frames centered on the peak LV intensity. The final myocardium was selected as the pixels agreed upon by 4/5th of the images. Their work, however, did not present an automated method for sector definition. Validation tests on 16 perfusion series gave similar regional flow indices to manual segmentations, but the spatial agreement of the segmentations was not reported. Hautvast *et al*.’s method [28] employed Otsu thresholding [29] of temporal projections of the image series to locate the heart region. The myocardium was segmented via a Hankel transform-based ring detector in the perfusion images. Tests on 25 perfusion studies comparing region-wise MBF values derived from time signal-intensity curves of manual and automatically segmented regions gave an *R*^2^ value of 0.805.

In addition to focusing on the perfusion images rather than the maps, all of these methods differed from our work in a few key ways. First, Gupta *et al*. [24], Adluru *et al*. [27], and Hautvast *et al*. [28] did not automatically segment or validate their methods on the apical slice. Second, none of the methods provided automatic segmental analysis. Tarroni *et al*. [25], Beache *et al*. [26], Adluru *et al*. [27], and Hautvast *et al*. [28] presented segmental analysis results using manually selected RV insertion point and sectors, while Spreeuwers and Breeuwer [23] and Gupta *et al*. [24] did not perform segmental analysis. Third, Tarroni *et al*.’s method [25] required user interaction for the myocardial segmentation. These omissions demonstrate both the difficulty and the need for fully automated segmental analysis.

As previously mentioned in the Introduction, two recent studies have utilized deep learning-based methods for myocardial segmentation of CMR perfusion pixel maps [13], [14]. The similarity of these works to our own methods warrants some comparison. Scannell’s work [13] provided independent test results based on 60 scans from 30 subjects for each of the four steps in their method, while Xue’s work [14] presented testing results of 200 scans from 105 patients for myocardial, LV, RV, and RV insertion point detection, and the resulting MBF measurements. The results from our conventional computer vision approach were measured on 486 scans from 243 studies. Here we will focus the comparison of our results with these methods based on 1) myocardial segmentation and Bland Altman analysis of measured MBF values in all three postposed methods, 2) the resulting sector-wise analysis with [13], and 3) RV insertion point accuracy with [14]. As Xue and our methods detect an RV insertion point angle, instead of the exact pixel location as in [13], a comparison of Scannell’s RV insertion point was not deemed practical. Similarly, Xue’s segmental analysis did not provide enough information to determine differences between automated and manual MBF measurements. They only reported that “the per-sector measures showed no difference between the CNN and manual measures (*P* = 0.92)” [14], so a direct MBF comparison in automatic vs. manual measured segments is not possible.

In our comparison with [13], [14], we find that 1) in the overall myocardial segmentation indices comparison, Scannell’s method achieved an average Dice score of 0.80 ± 0.06 over all slices, compared to the proposed method’s 0.86 ± 0.06 and Xue’s 0.93 ± 0.04. Scannell’s Bland Altman analysis yielded a bias of 0.04 ml/g/min versus the proposed methods 0.01 ml/g/min. Xue’s paper did not report exact bias numbers, but their Bland Altman plot shows a bias less than 0.025 ml/min/g. Scannell did not report segmentation failure on any cases, while Xue and the proposed method reported two failed segmentations each. 2) Scannell’s sector-wise analysis showed an *R*^2^ of 0.76 over all three slices while our results achieved 0.94. 3) Xue’s RV insertion point differed from the manual by 2.65 ± 3.89°, compared with the proposed method’s 3.19 ± 4.16°. Over all these comparisons show that the three methods provide similar results of comparable segmentation performance; however, in general the proposed method yields slightly superior results to Scannell *et al*. [13] but slightly inferior results to Xue *et al*. [14].

In terms of methodology, the main differences between Scannell and Xue’s methods versus our approach are 1) the use of deep learning methods at each stage of processing, and 2) the decision to use the perfusion image myocardial segmentation to limit both quantification and segmental analysis. Regarding the former, deep learning is a burgeoning segment of the medical image processing field and is likely to result in great advances. Its main drawback is the requirement for large amounts of independently and manually generated training data; hence why 135 of Scannell’s 175 subjects and 1825 scans Xue’s dataset were relegated to the training dataset. Due to the present rarity of such large, labeled datasets, the proposed method opted for more traditional image processing techniques and was able to maintain a similar performance.

Regarding the latter, in contrast to Scannell’s, Xue’s and other previous methods, the proposed automated processing method includes segmentation of the myocardium from both the myocardial perfusion images and the MBF map images. This is implemented by using an initial myocardial segmentation from the perfusion images to refine the myocardial segmentation of the MBF map. From our experience, myocardial segmentation performed on either perfusion image series or MBF maps alone had limitations. By leveraging the information from the perfusion images and MBF maps together, a more reliable and robust segmentation was achieved.

For example, residual motion, partial volume effects, and perfusion imaging artifacts may hamper the accuracy of the myocardial segmentation in the perfusion images. These issues could introduce mismatched myocardial borders frame-to-frame within the image series and hinder the consistency of the myocardial region between the image series and the MBF maps. On the other hand, segmentation of the RV and LV regions can be more reliably performed on the perfusion images due to distinct time-varying contrast signal in the blood pool.

In contrast, myocardial segmentation performed on the MBF maps alone also introduced technical difficulties. For example, the background region in the maps appears patchier and noisier than the perfusion images (the result of quantifying non-myocardial pixels), which can make it more difficult to isolate the epicardial edge than in the perfusion images. However, MBF map segmentation is more robust to the residual motion and blurring effects after the deconvolution step as it provides a low-pass smoothing effect to the pixel time-signal intensity curves. It also produces a more practical MBF value to help differentiate the myocardial pixels, rather than using arbitrary signal intensity units. By segmenting the perfusion images first, we can better constrain the segmentation in the MBF maps to remove the background non-myocardial pixels and locate the myocardial pixels, making the overall method more robust and accurate.

Despite the similarity of these works, each still stands on its own as worthwhile, complementary processes, and none should invalidate the others. The inline approach by Xue *et al*. [14], for example may increase speed of initial data availability, while the proposed post-processing methods allow re-analysis of legacy data, or modification of segmentation or quantification parameters for different acquisition protocols or non-typical cases.

The presented work has some limitations. Our primary dataset in the current study is limited to scans from one center, and two scanners, and all use the same dual-sequence imaging protocol. That said, we include in the appendix two smaller independent validation datasets, one from our institution but using an alternate imaging protocol, and another from an external institution. Further, while the presented automated MBF map segmentation does not depend on one specific AIF imaging protocol, a dedicated AIF acquisition method (such as the dual-sequence [15] or the dual-bolus approach [30]) is required for the MBF maps generation, but these methods are not yet widely available clinically. However, it is hoped the results presented will bolster the use of quantitative CMR perfusion in the field.

It is worth noting that this limitation is also shared by the discussed deep learning methods. The current deep learning methods have not been shown to be generalizable to different acquisition protocols. While Scannell’s study only used dual-bolus perfusion acquisition [13], Xue’s approach only worked in dual-sequence perfusion imaging [14]. There is a need for a direct comparison of different perfusion quantification and segmentation approaches based on a common and large dataset that includes multi-centers, multi-vendors, and multi-acquisition protocols to evaluate their clinical diagnostic performance. It is also our hope that increased data availability and diversity will improve all AI methods to be generalizable to different environments and diseases in the near future.

Since the goal of the presented work was to perform myocardial segmentation on the MBF maps, as opposed to the perfusion image segmentation methods surveyed earlier, we did not perform independent validation for the myocardial segmentation on the perfusion image series. In the proposed method, the perfusion image segmentation is only an intermediate step to improve the accuracy of the final MBF map segmentation. It is therefore less important and unclear whether the intermediate segmentation of the perfusion images is accurate enough to justify its use in separate applications. However, future work could test the accuracy of this step in more detail and perhaps improve upon both it and the MBF map segmentation. Similarly, it is acknowledged that individual processing steps in generating the perfusion pixel maps, such as motion correction or deconvolution, may affect the MBF map segmentation. Nevertheless, it is beyond the scope of this work to evaluate the effects of each of these individual steps for the final segmentation accuracy.

Finally, our evaluation makes use of an 18-sector model based on one RV insertion point at the basal slice instead of a more common AHA 16-sector model. However, given the same model was used for both manual and automatic methods, it should not affect the overall conclusions of the study. Furthermore, this was not an imminent concern because mapping the sector-wise MBF measurements to arterial territories (the primary utility of AHA segmental analysis) can be reprogrammed according to different needs.

## V. CONCLUSION

We have presented a fully automated method for segmental analysis of the myocardium from CMR imaging perfusion MBF maps. The method successfully processed 243 of 247 clinical studies. Further, the proposed method was shown in excellent agreement with a manual reference standard in terms of segmented area and region derived perfusion measurements. The presented method will be integrated with our fully automated CMR perfusion pixel mapping pipeline to aid in its use and proliferation for different clinical applications, as well as future research into improved methods for computer aided diagnosis.

## Figures and Tables

**TABLE 1.** Patient demographic data.

Gender (male: female)	162:85
Age (year)	50 ± 18
Weight (kg)	82 ± 19
Height (cm)	171 ± 12
Body Mass Index (kg/m^2^)	28.1 ± 5.7
Body Surface Area (m^2^)	1.9 ± 0.2
Baseline Heart Rate (beats/min)	65 ± 12
Peak Heart Rate (beats/min)	98 ± 16
Baseline Systolic Blood Pressure (mmHg)	128 ± 19
Peak Systolic Blood Pressure (mmHg)	124 ± 20
Baseline Diastolic Blood Pressure (mmHg)	75 ± 14
Peak Diastolic Blood Pressure (mmHg)	72 ± 15

**TABLE 2.** Myocardial area agreement metrics.

	Basal	Mid	Apical	Total
Dice	0.86 ± 0.05	0.88 ± 0.05	0.84 ± 0.08	0.86 ± 0.06
Cohen Kappa	0.86 ± 0.05	0.87 ± 0.05	0.84 ± 0.08	0.86 ± 0.06

**TABLE 3.** Myocardial border distance metrics.

(mm)	Basal	Mid	Apical	Total
Epicardial	1.34 ± 0.56	1.52 ± 0.81	1.56 ± 0.77	1.47 ± 0.73
Endocardial	0.95 ± 0.46	1.13 ± 0.54	0.99 ± 0.50	1.02 ± 0.51

**TABLE 4.** Myocardial area agreement metrics.

	Rest	Stress	Total
Dice	0.86 ± 0.06	0.85 ± 0.06	0.86 ± 0.06
Cohen Kappa	0.86 ± 0.05	0.85 ± 0.06	0.86 ±0.06

**TABLE 5.** Myocardial border distance metrics.

(mm)	Rest	Stress	Total
Epicardial	1.34 ± 0.66	1.59 ± 0.77	1.47 ± 0.73
Endocardial	0.93 ± 0.40	1.11 ± 0.58	1.02 ± 0.51

**FIGURE 8.**
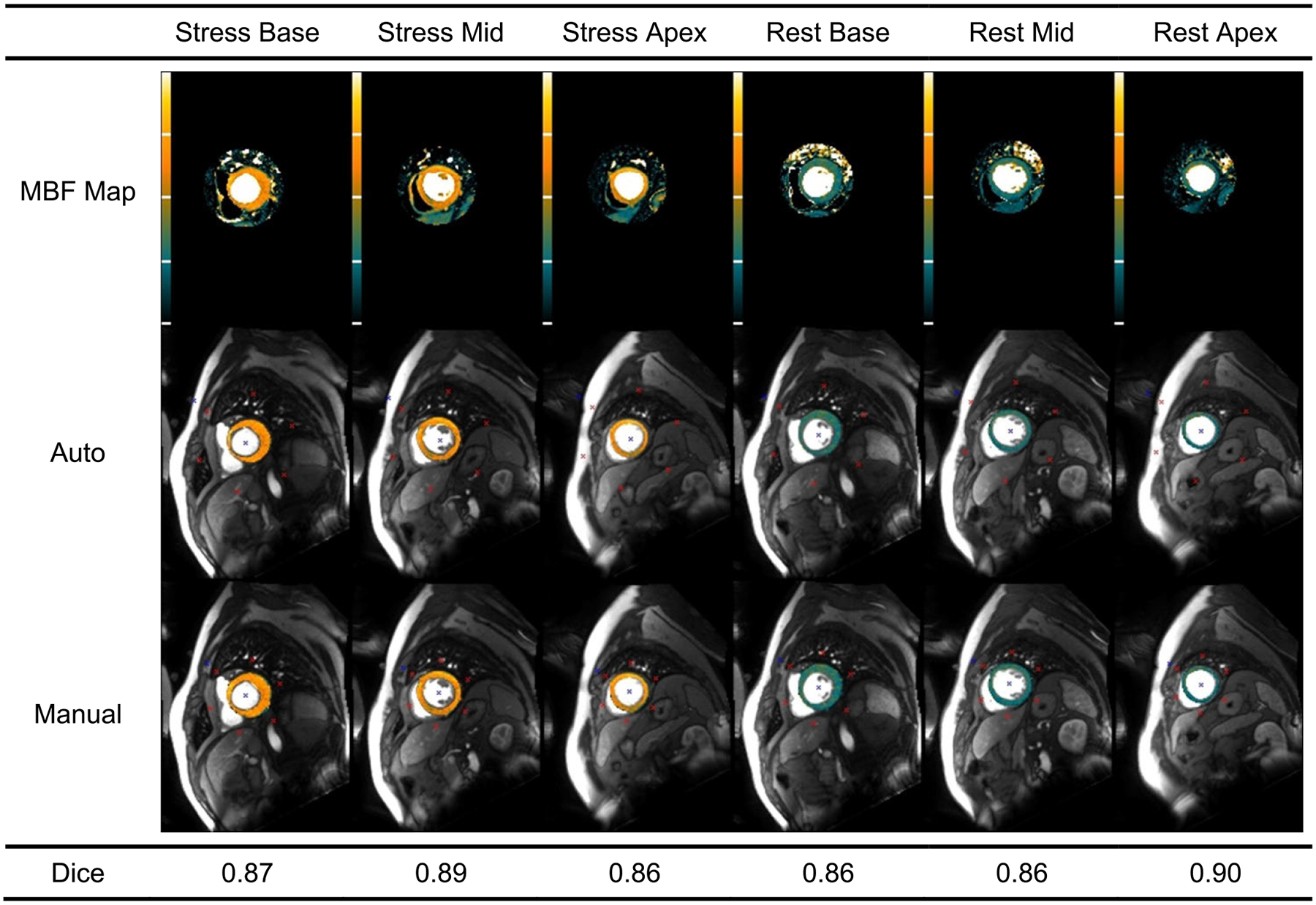
Dual bolus example result: Comparison of automated and manual segmentation of stress and rest MBF maps obtained from a different imaging protocol based on dual bolus acquisition approach.

**FIGURE 9.**
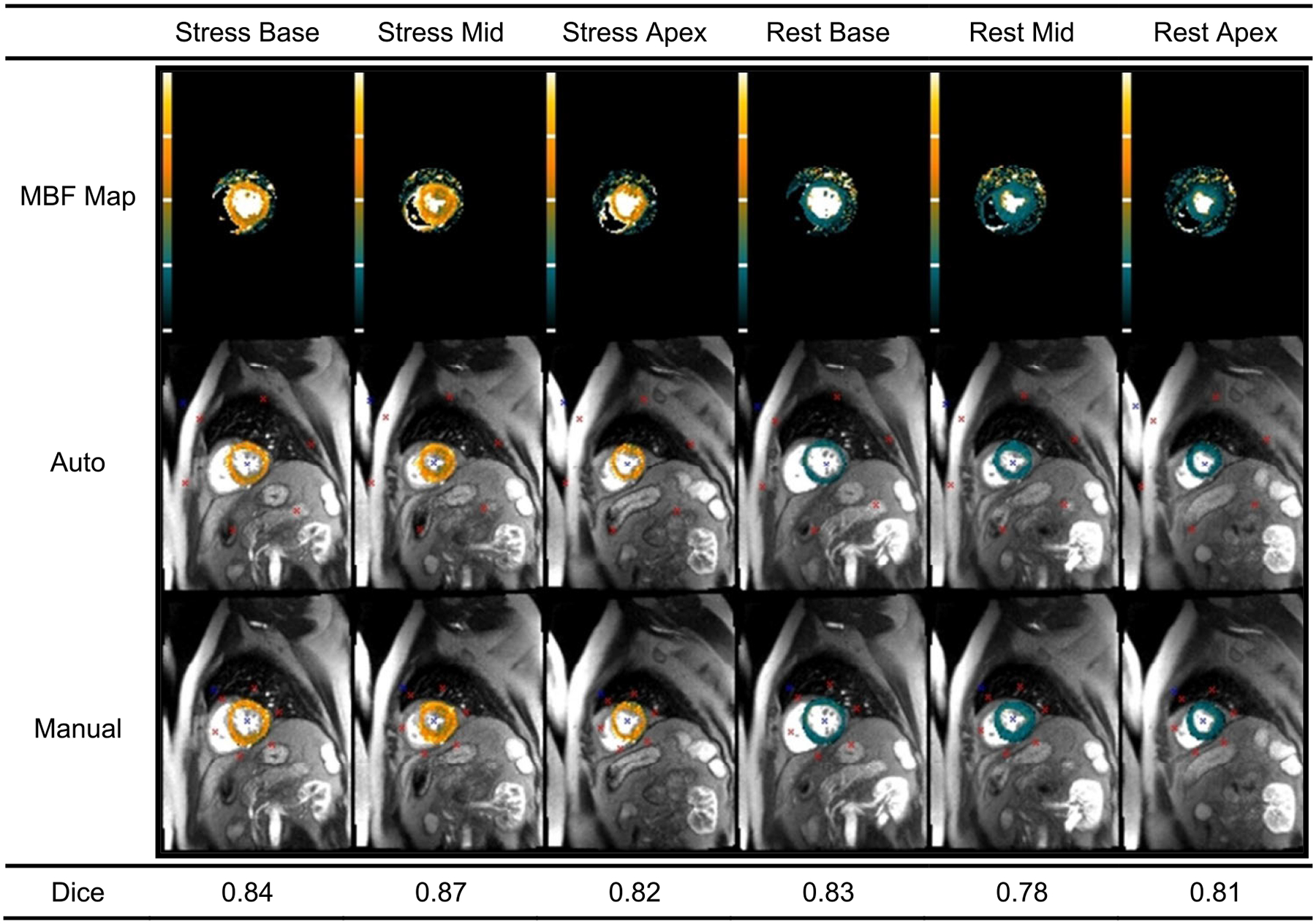
Alternate institution example result: Comparison of automated and manual segmentation of stress and rest MBF maps obtained from an independent institution using a different scanner and imaging parameters.

## APPENDIX A

We have assembled a secondary, independent dataset acquired in our institution which makes use of a different acquisition protocol: dual bolus AIF acquisition scheme, details of which are described in [3], rather than the dual sequence method in our primary dataset. These additional tests are intended to determine the generalizability of our approach to different acquisition protocols.

This set of independent data is composed of 25 dual bolus perfusion studies that have adequate vasodilator response for quantitative CMR perfusion imaging and MBF quantification. They include the same three slices at rest and stress as our primary dataset. All patients have signed research consent documents and all data was fully anonymized to meet our institutional requirements for patient inclusion before image processing.

The MBF maps of these cases were manually contoured, and then compared with the segmentation generated by the proposed automated method. The results of this independent dual bolus dataset are summarized in Table 6 and Table 7. Comparison of sector-wise perfusion values between automated and manual segmental analyses yielded Pearson’s correlation coefficients of 0.98 (*p* < 0.001) for MBF estimates, and 0.89 (*p* < 0.001) for MPR estimates. An example of our automated segmentation is included in the Figure 8.

**TABLE 6.** Myocardial area agreement metrics.

	Basal	Mid	Apical	Total
Dice	0.86 ± 0.05	0.86 ± 0.06	0.83 ± 0.10	0.85 ± 0.07
Cohen Kappa	0.86 ± 0.05	0.86 ± 0.06	0.83 ± 0.10	0.85 ± 0.08

**TABLE 7.** Myocardial border distance metrics.

(mm)	Basal	Mid	Apical	Total
Epicardial	1.57 ± 0.52	1.75 ± 1.01	1.93 ± 1.75	1.75 ± 1.22
Endocardial	0.79 ± 0.37	1.10 ± 0.63	0.83 ± 0.62	0.91 ± 0.57

**TABLE 8.** Myocardial area agreement metrics.

	Basal	Mid	Apical	Total
Dice	0.80 ± 0.04	0.84 ± 0.04	0.78 ± 0.07	0.81 ± 0.06
Cohen Kappa	0.80 ± 0.04	0.84 ± 0.04	0.77 ± 0.07	0.80 ± 0.06

**TABLE 9.** Myocardial border distance metrics.

(mm)	Basal	Mid	Apical	Total
Epicardial	1.88 ± 0.41	2.12 ± 0.63	2.00 ± 0.51	1.99 ± 0.54
Endocardial	1.25 ± 0.49	1.09 ± 0.47	1.21 ± 0.68	1.18 ± 0.56

Analysis shows that these dual bolus results are comparable to our primary results from the dual sequence dataset presented in the body of paper, helping to validate our methods’ generalizability to alternative quantitative perfusion imaging approaches.

## APPENDIX B

We have assembled a tertiary, independent dataset consisting of scans performed at a partnered institution (NHS Golden Jubilee National Hospital, Clydebank, United Kingdom) using a different scanner running different imaging parameters than those used in our institution. These additional tests are intended to determine the generalizability of our approach to different scanners and imaging parameters.

This set of independent data is composed of 25 perfusion studies captured using a dual sequence acquisition method, similar to our primary dataset, which includes both rest and stress scans of three slices. All images were fully anonymized before reaching our institution to meet the source institution’s requirements for data sharing. All studies had adequate vasodilator response for quantitative CMR perfusion imaging and MBF quantification. However, the perfusion images in this test dataset had noticeably lower spatial resolution and more pronounced image noise than the data from our institution.

The MBF maps of these studies were manually contoured and then compared with the proposed automated segmentation method. The results of this independent dual sequence dataset are summarized in Table 8 and Table 9. Comparison of sector-wise perfusion values between automated and manual segmental analyses yielded Pearson’s correlation coefficients of 0.95 (*p* < 0.001) for MBF estimates, and 0.78 (*p* < 0.001) for MPR estimates. An example of our automated segmentation is shown in Figure 9

Compared with the results of the primary data from our institution, these quantitative segmentation metrics show a slightly lower Dice score and slightly increased distance measurement from this independent dataset. However, this small performance decrease is not surprising due to the lower resolution imaging setting used at this clinical site, which resulted in larger myocardial pixels in the images and led to greater distance errors. Despite this, the overall agreement metrics are still acceptable in the basal and mid slices. Only the apical slice suffers more considerably, perhaps due to partial volume effect as well as motion artifacts. This additional analysis helps validate our method’s generalizability to external datasets collected from independent clinical sites with difference scanning configurations.

## APPENDIX C

In addition to the quantitative metrics presented in Tables 2 to 9, several supplementary metrics were assessed to evaluate the overall segmentation performance. These include accuracy, sensitivity, specificity, positive predictive value (PPV, also referred to as precision), volume overlap error (VOE), relative volume difference (VD) [22], and the border distance metrics of the average symmetric surface distance (ASSD) [22], Hausdorff distance (HD), and Mahalanobis distance (MD). Table 10 presents the assessment results of our primary dataset. Table 11 summarizes the results of the independent dataset in Appendix A, while Table 12 gives the results of the independent dataset in Appendix B.

Please note that one reason these metrics were not included in the body of the paper is due to known biases in some of them. For example, specificity is the ratio of pixels classified as true negative (correct background) to all true negative and false positive pixels. Due to a relatively small myocardial region of interest in our images, the vast number of true negative pixels of the background region overwhelms the relatively few false positive pixels, resulting in a misleadingly high metric.

**TABLE 10.** Additional assessment metrics for the primary dataset (N = 243 subjects, 1458 slices).

Accuracy (%)	Sensitivity (%)	Specificity (%)	PPV (%)	VOE (%)	VD (%)	ASSD (mm)	HD (mm)	MD (mm)
99.48 ± 0.21	87.87 ± 09.92	99.65 ± 0.19	78.36 ± 9.70	29.56 ± 10.00	8.21 ± 6.84	1.28 ± 0.60	14.27 ± 3.70	0.173 ± 0.12

**TABLE 11.** Additional assessment metrics for the independent dataset in Appendix A (N = 25 subjects, 150 slices).

Accuracy (%)	Sensitivity (%)	Specificity (%)	PPV (%)	VOE (%)	VD (%)	ASSD (mm)	HD (mm)	MD (mm)
99.49 ± 0.25	89.48 ± 10.14	99.64 ± 0.18	77.55 ± 8.55	29.26 ± 9.76	9.49 ± 7.05	1.36 ± 0.72	13.97 ± 3.77	0.19 ± 0.12

**TABLE 12.** Additional assessment metrics for the independent dataset in Appendix B (N = 25 subjects, 150 slices).

Accuracy (%)	Sensitivity (%)	Specificity (%)	PPV (%)	VOE (%)	VD (%)	ASSD (mm)	HD (mm)	MD (mm)
99.41 ± 0.16	83.05 ± 9.51	99.66 ± 0.15	79.10 ± 9.76	31.70 ± 10.62	5.87 ± 4.53	1.59 ± 0.38	12.75 ± 3.30	0.23 ± 0.14
